# Effect of Excitation Signal on Double-Coil Inductive Displacement Transducer

**DOI:** 10.3390/s23073780

**Published:** 2023-04-06

**Authors:** Yanchao Li, Ruichuan Li, Junru Yang, Jikang Xu, Xiaodong Yu

**Affiliations:** 1 College of Mechanical and Electronic Engineering, Shandong University of Science and Technology, Qingdao 266590, China; 202081050050@sdust.edu.cn (Y.L.);; 2 College of Transportation, Shandong University of Science and Technology, Qingdao 266590, China

**Keywords:** double-coil inductive displacement transducer, transducer characteristics, conditioning circuit, excitation signal, linearity, sensitivity

## Abstract

A double-coil inductive displacement transducer is a non-contact element for measuring displacement and is widely used in large power equipment systems such as construction machinery and agricultural machinery equipment. The type of coil excitation signal has an impact on the performance of the transducer, but there is little research on this. Therefore, the influence of the coil excitation signal on transducer performance is investigated. The working principle and characteristics of the double-coil inductive displacement transducer are analyzed, and the circuit simulation model of the transducer is established. From the aspects of phase shift, linearity, and sensitivity, the effects of a sine signal, a triangle signal, and a pulse signal on the transducer are compared and analyzed. The results show that the average phase shift, linearity, and sensitivity of the sine signal were 11.53°, 1.61%, and 0.372 V/mm, respectively; the average phase shift, linearity and sensitivity of the triangular signal were 1.38°, 1.56%, and 0.300 V/mm, respectively; and the average phase shift, linearity, and sensitivity of the pulse signal were 0.73°, 1.95%, and 0.621 V/mm, respectively. It can be seen that the phase shift of a triangle signal and a pulse signal is smaller than that of a sine signal, which can result in better signal phase-locked processing. The linearity of the triangle signal is better than the sine signal, and the sensitivity of the pulse signal is better than that of the sine signal.

## 1. Introduction

At present, displacement measurement systems are ubiquitous and mainly divided into direct and indirect measurements. Direct measurement includes laser interferometers, grating rulers, laser rangefinders, capacitive sensors, inductive sensors, and ultrasonic sensors. Indirect measurement includes three-dimensional coordinate machines, visual measurement, speed sensors, and acceleration sensors. The laser interferometer can measure displacement at the wavelength level, and the laser rangefinder is suitable for long-distance measurement. Capacitive and inductive sensors are suitable for small displacement and high-precision measurements. The inductive displacement sensors are non-contact measurements with higher reliability.

The inductive transducer is a device that uses the change in self-inductance or mutual inductance of inductance coils to realize measurement. It can measure displacement, pressure, angle, speed, flow, and other parameters [1], which are widely used in high-end manufacturing equipment [2], the automotive industry [3], agricultural machinery equipment, and construction machinery. Inductive transducers have the characteristics of high reliability, high sensitivity, and good linearity, which can achieve non-contact measurement and are suitable for working in dusty, oily, mechanical vibration, and other harsh environments [4,5].

There are many types of inductive displacement transducers, which are mainly divided into three types: self-inductive displacement transducers [6], mutual inductance displacement transducers [7], and eddy current displacement transducers [8]. Different types of displacement sensors have different applications, but the working principle is the same, that is, the measured displacement is converted into the change of inductance by electromagnetic induction, and then the change of inductance is converted into the change of voltage or current by conditioning circuits to achieve non-electric to electric conversion. In precision measurement systems, the performance of inductive displacement transducers significantly impacts measurement accuracy [9]. Therefore, it is important to study inductive displacement transducers with good linearity, high resolution, and high accuracy [10].

To improve the performance of inductive displacement transducers and reduce production costs, researchers in the field of transducers have conducted a lot of work. In order to improve the measurement accuracy of the inductive angle displacement transducer, Gao WZ et al. designed a non-contact planar inductive angle displacement transducer. The conditioning circuits of the transducer use an FPGA chip, and the FPGA outputs two pulse signals, one of which is converted into a sine signal as an excitation signal for the coil, and the other signal is a modulation signal. The transducer is suitable for high-precision, reliable measurements in harsh environments. However, it is necessary to convert the pulse signal into a sine signal for coil excitation, which increases the complexity of the conditioning circuit [11]. The conditioning circuits of traditional LVDT transducers use integrated chips to realize signal processing, and in order to reduce costs, low-cost electronic components can be used to build demodulation circuits, but the reliability of the circuit will be reduced [12]. The coil excitation of an inductive transducer is an AC signal, and the type, amplitude, and frequency of the excitation signal have a certain influence on the performance of the transducer. Sever PS studied AC signal generation circuits that used Colpitts or Hartley oscillators to generate sine signals of constant frequency and amplitude to achieve coil excitation of inductive displacement transducers [13]. For dual excitation and dual induction coil displacement transducers, Wu XW studied the effect of excitation frequency on sensitivity and determined the excitation frequency of the highest sensitivity. However, the magnitude and type of excitation have not been studied [14]. The zero drift of the inductive transducer is one of the indicators to measure its performance. The bridge differential inductance detection circuit can reduce the zero drift. For an inductive distance transducer, Zhao YY proposed an automatic zero-adjustment technology based on a PID controller by using sine signals as coil excitation, which realized the consistency of production products and improved the efficiency of installation and debugging [15]. The output signal of an inductive transducer is generally an analog signal, and the coupling capacitance has a certain influence on the output signal of transducers during the working process. A successive approximation signal conditioning circuit enables the transducer to output a digital signal. However, digital transducers increase production costs and are cumbersome to install and debug [16]. In terms of inductive transducer structure, the accuracy and sensitivity of output signals can be improved by improving the core end structure and changing the magnetic field distribution [17,18]. By adding a magnetic field shield structure, external interference can be reduced, and the accuracy of the output signal can be improved [19,20].

It can be seen from the above that in order to optimize the performance of inductive displacement transducers, the frequency of the excitation signal, the structure of the transducer, external interference, and the conditioning circuit are studied. The above research results improve the linearity, sensitivity, and accuracy of inductive displacement transducers to varying degrees and improve the measurement accuracy of transducers. However, we have not seen studies on the types of excitation signals of inductive displacement transducers. At present, the commonly used coil excitation signal is a sine signal, but microprocessor chips (such as STM32 processor chips) generally output a pulse signal, and a conversion circuit needs to be added to convert the pulse signal into a sine signal. In order to reduce signal processing links and production costs, it is necessary to find a simple and reliable coil excitation signal.

Based on the double-coil inductive displacement transducer, this paper describes the structure and working principle of the double-coil inductive displacement transducer establishes a simplified model of the inductance coil and analyzes the output characteristics of the transducer. The inductance coils are connected by a differential bridge, and a conditioning circuit based on the AD630 chip is designed to process the original signal of the transducer. Based on the AD630 conditioning circuit, the demodulation principles of three excitation signals are clarified. Using Multisim software, the simulation model of the conditioning circuit was established, and the linearity and sensitivity of the transducer were analyzed and compared with the traditional sine excitation signal. Finally, experimental verification is carried out, which proves the effectiveness of the excitation signal type proposed in this paper.

## 2. Structure and Working Principle of Double-Coil Inductive Displacement Transducer

### 2.1. Structure of Displacement Transducer

**Figure 1:**
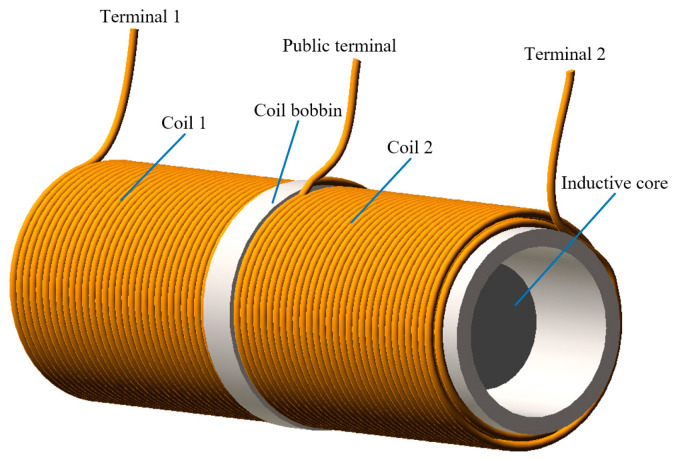
Structure diagram of a double-coil inductive displacement transducer.

The structure diagram of the double-coil inductive displacement transducer is shown in Figure 1. The transducer is composed of two inductance coils in series, a skeleton, and a sensing core. Inductance coils are made of copper wire, and the structure of both coils is identical. The two coils have a total of three terminals. The connection node of the two coils is the common terminal, and the other ends of the coil are terminals 1 and 2, respectively. The skeleton is used to support the coil, which is made of epoxy resin with a magnetic insulation effect. The sensing core has good magnetic conductivity and is made of silicon steel sheet material. There is a certain gap between the sensing core and the inner diameter of the skeleton, which can realize the non-contact measurement of displacement.

### 2.2. Working Principle of Displacement Transducer

When an AC excitation signal is applied to the double-coil inductive displacement transducer, an alternating magnetic field is generated around the coil, and the distribution of the magnetic field will affect the inductance of the coil.

The calculation formula for coil inductance is [21]

(1) $$L=\frac{\Phi }{I}=\frac{a\int BdS}{I}=\frac{a\int \mu nIdS}{I}=\frac{a\mu nIS}{I}=a\mu nS$$

where *L* is the coil inductance, Φ is the coil flux, *a* is the coil length, *n* is the number of coil turns per unit length, *I* is the current flowing through the coil, *S* is the cross-sectional area of the coil, *B* is the magnetic field strength in the coil, and *μ* is the magnetic permeability.

According to Equation (1), it can be seen that the inductance of the double-coil displacement transducer is related to the inner magnetic permeability of the coil. The working essence of the double-coil inductive displacement transducer is that the sensing core’s motion changes the magnetic circuit’s reluctance so that the coil’s inductance changes. The conditioning circuits convert changes in inductance into changes in voltage or current and determine the displacement by measuring the changes in voltage or current.

**Figure 2:**
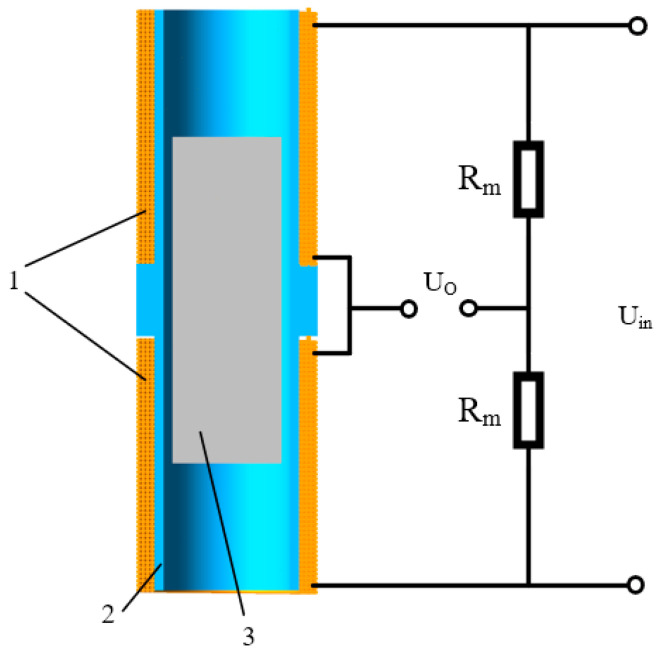
Working principle of the double-coil inductive displacement transducer. (1) Coil, (2) skeleton, and (3) sensing core.

The working principle of the double-coil inductive displacement transducer is shown in Figure 2. The two coils are connected by a bridge [22,23,24], with R_m_ as the matching resistor of the bridge circuit, U_in_ as the excitation voltage, and Uo as the output signal. In the initial state, the sensing core is in the middle position, and the length of the sensing core inserted into the two coils is equal. The magnetic permeability of coils 1 and 2 is equal, so the inductance of the two coils is equal. At this point, U_o_ = 0. When the sensing core is off-center and moves up or down, the magnetic resistance of the two coils changes so that the inductance of the two coils increases and decreases. At this time, the bridge circuit is unbalanced. The amplitude of the output voltage of the bridge circuit is proportional to the movement of the sensing core, and its phase is related to the direction of the movement of the sensing core. If the sensing core moves downward, the output voltage is positive; if the sensing core moves upward, the output voltage is negative. Therefore, as long as the magnitude and phase of the output voltage can be measured, the magnitude and direction of the sensing core displacement can be determined. The sensing core is rigidly connected to the measured object, and the displacement of the sensing core is equal to the measured object.

## 3. Electromagnetic and Electrical Characteristics of Double-Coil Inductive Displacement Transducers

### 3.1. Electromagnetic Characteristics of Inductance Coil

**Figure 3:**
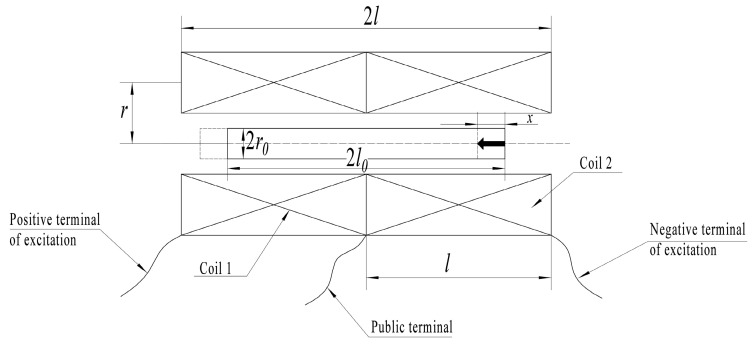
Structure parameters of the coil.

The displacement of the sensing core changes, and the magnetoresistance of the magnetic circuit changes. The inductance of the coil is related to magnetoresistance. The precise theoretical analysis of such transducers is complicated by the uneven distribution of axial magnetic field intensity along finite-length coils. The structural parameters of the coil are shown in Figure 3. *l* is the length of the single coil, *r* is the radius of the coil, *l*_0_ is half the length of the sensing core, *r*_0_ is the radius of the sensing core, and *x* is the displacement of the sensing core.

In order to simplify the analysis, set the length-to-diameter ratio of the coil to *l*/*r* ≫ 1, then the magnetic field intensity in the coil can be considered to be evenly distributed. The magnetic field intensity *H* at the center of the coil is

(2) $$H=\frac{IN}{l}$$

The magnetic induction intensity *B* is

(3) B=μH

The inductance of the hollow core coil is [25]

(4) $$L_{0}=\frac{\Psi }{I}=\frac{N\times \Phi }{I}=\frac{N\times B\times S}{I}=\frac{\mu_{0}N^{2}\pi r^{2}}{l}$$

where *Ψ* is the linkage, Φ is the magnetic flux, and μ_0_ is the permeability in the hollow coil.

After the sensing core is inserted into the coil, because the sensing core is a magnetically permeable material, the magnetoresistance of the inserted part decreases because the sensing core is a magnetic material. Therefore, the magnetic induction intensity *B* increases, and the inductance increases.

After inserting the sensing core, the total magnetic flux Φ_a_ of the coil and the inductance *L* of the coil are [26,27]

(5) $$\Phi_{a}=\mu_{0}\mu_{m}H\pi r_{0}{}^{2}+\mu_{0}H\pi (r^{2}-r_{0}{}^{2})=\mu_{0}H\pi \left[(\mu_{m}-1)r_{0}{}^{2}+r^{2}\right]$$

(6) $$L=\frac{N\Phi_{a}}{I}=\frac{\mu_{0}\pi N^{2}\left[(\mu_{m}-1)r_{0}{}^{2}+r^{2}\right]}{l}$$

Since the length of the sensing core is less than the coil [28], the inductance *L* of the coil is

(7) $$L=\frac{\mu_{0}\pi N^{2}\left[(\mu_{m}-1)l_{0}r_{0}{}^{2}+lr^{2}\right]}{l^{2}}$$

When *l*_0_ increases by *x*, the coil inductance increases by ∆*L*, then

(8) $$L+\Delta L=\frac{\mu_{0}\pi N^{2}\left[(\mu_{m}-1)(l_{0}+x)r_{0}{}^{2}+lr^{2}\right]}{l^{2}}$$

The variation of inductance is

(9) $$\Delta L=\frac{\mu_{0}\pi N^{2}r_{0}{}^{2}(\mu_{m}-1)x}{l^{2}}$$

As can be seen from Equation (9), ∆*L* is proportional to *x*.

### 3.2. Electrical Characteristics of Inductance Coils

**Figure 4:**
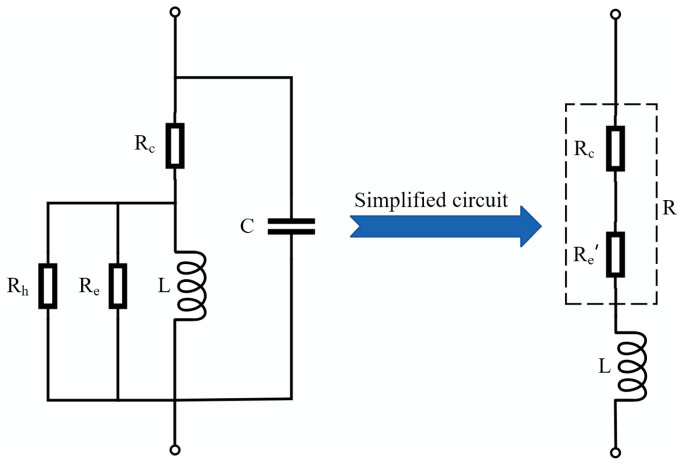
Simplified model of the inductance coil.

The inductance coil is not an ideal pure inductive element; in addition to the inductor *L*, it also includes the copper loss resistance *R_c_*, the core eddy current loss resistance *R_e_*, the hysteresis loss resistance *R*_h_, and the parallel parasitic capacitance *C*. In order to simplify the analysis, the parallel parasitic capacitance is ignored. *R_e_* and Rh are equivalent to the resistor *R_a_* in series with the inductance coil, and *R_c_* and *R*_a_ are equivalent to *R*. A simplified model of the inductance coil is shown in Figure 4 [29,30,31].

**Figure 5:**
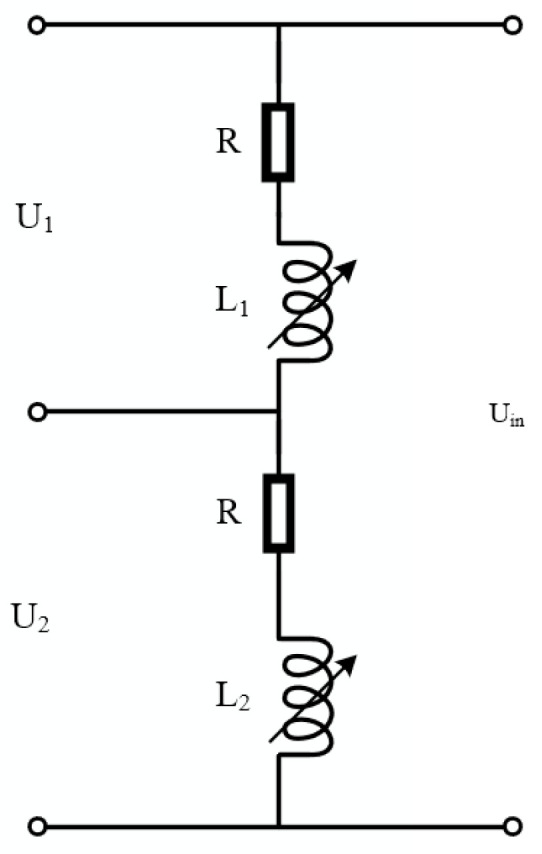
Equivalent circuit of a double-coil inductive displacement transducer.

The equivalent circuit of the double-coil inductive displacement transducer is shown in Figure 5 [32]. L_1_ and *L*_2_ are the inductances of coils 1 and 2, and the voltages of coils 1 and 2 are U_1_ and U_2_, respectively.

The impedances *Z*_1_ and *Z*_2_ of coils 1 and 2 can be expressed as

(10) $$Z_{1}=R+j\omega L_{1}$$

(11) $$Z_{2}=R+j\omega L_{2}$$

When the sensing core is in the middle position, *L* = *L*_1_ = *L*_2_, and the voltage of the two coils is equal, that is

(12) $$U_{1}=U_{2}=\frac{U_{in}}{2}$$

When the sensing core deviates from the middle position, the inductance of both coils changes. If the inductance of coil 1 increases by ∆*L*, the inductance of coil 2 decreases by ∆*L*. The impedances of coils 1 and 2 are

(13) $$Z_{1}=R+j\omega (L_{1}+\Delta L)$$

(14) $$Z_{2}=R+j\omega (L_{2}-\Delta L)$$

At this point, the voltages of coils 1 and 2 are

(15) $$U_{1}=\frac{Z_{1}}{Z_{1}+Z_{2}}U_{in}=\frac{R+j\omega (L_{1}+\Delta L)}{2(R+j\omega L)}U_{in}=\frac{U_{in}}{2}+\frac{j\omega \Delta L}{2(R+j\omega L)}U_{in}$$

(16) $$U_{2}=\frac{Z_{2}}{Z_{1}+Z_{2}}U_{in}=\frac{R+j\omega (L_{2}-\Delta L)}{2(R+j\omega L)}U_{in}=\frac{U_{in}}{2}-\frac{j\omega \Delta L}{2(R+j\omega L)}U_{in}$$

The voltage variation ∆*U* is

(17) $$\Delta U=\frac{j\omega \Delta L}{2(R+j\omega L)}U_{in}$$

As can be seen from Equation (17), ∆*U* is proportional to ∆*L*. Because ∆*L* is proportional to *x*, ∆*U* is proportional to *x*.

## 4. Conditioning Circuit of Double-Coil Inductive Displacement Transducer

Double-coil inductive displacement transducers convert the displacement change of the measured object into a change in inductance. In order to measure the change in inductance, it is necessary to use a conditioning circuit to convert the change in inductance into a change in voltage or current. Generally, inductance can be converted into the amplitude, frequency, and phase of voltage or current. They are called amplitude modulation circuits, frequency modulation circuits, and phase modulation circuits, respectively. Based on the AD630 chip, a voltage amplitude modulation circuit is designed in this paper that converts the change in inductance into a change in voltage amplitude and realizes the change in sensing core displacement into a change in voltage amplitude.

**Figure 6:**
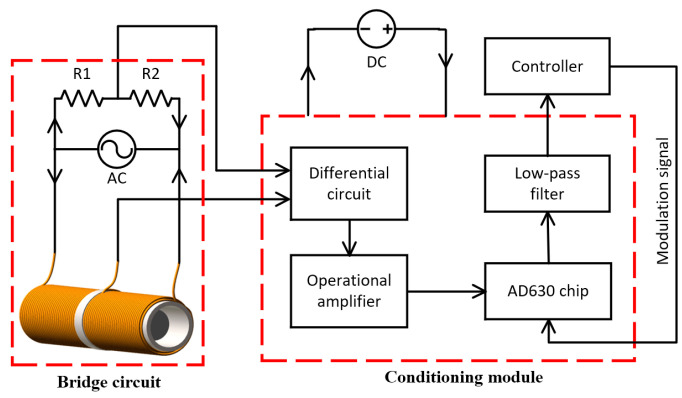
Working principle of the conditioning circuit.

The amplitude modulation circuit designed in this article uses a differential bridge connection, as shown in Figure 2. Since the coil excitation signal is an AC voltage signal, the output voltage Uo is also an AC signal. The function of the demodulation circuit is to convert a changing AC voltage signal into a changing DC voltage signal. The working principle of the conditioning circuit is shown in Figure 6. The conditioning circuit consists of a differential circuit, an operational amplifier circuit, an AD630 chip, and a low-pass filter [33].

First, the bridge circuit outputs the AC voltage signal to the differential circuit and performs a difference calculation on the voltage at the public terminal of the coil and the voltage at the public terminal of resistors R1 and R2. The differential output signal is then amplified by the operational amplifier circuit. Next, the AD630 chip is used to realize the phase-locked processing of the amplified signal, and the double-voltage AC voltage signal is converted into a single-voltage AC signal. Finally, the AC voltage signal with varying amplitude is converted into a changing DC voltage signal through a low-pass filter. The modulation signal of the AD630 is the pulse signal output by the controller.

## 5. Comparison of Three Excitation Signals

The excitation signal of the inductance coil is an AC signal, and the alternating current will generate a magnetic field around the coil. Changes in the sensing core will cause changes in the magnetic field, resulting in a change in inductance. At present, the commonly used AC signal is a sine signal. On the one hand, the excitation signal output by integrated demodulation chips in most transducers is a sine signal. On the other hand, the oscillation circuit is simple in design, and the sine signal can be generated by using inductance and capacitance. However, in practical applications, the microprocessor used by the controller generally cannot directly generate sine signals but can generate triangular signals and pulse signals, so it is necessary to add a sine signal generation circuit. In order to simplify the circuit, this paper proposes using the triangular signal or the pulse signal as the excitation signal of the inductance coil [34,35,36,37], analyzes the demodulation principle of the sine signal, triangle signal, and pulse signal, establishes the simulation circuit model, and compares the three excitation signals.

### 5.1. Demodulation Principle of Three Excitation Signals

**Figure 7:**
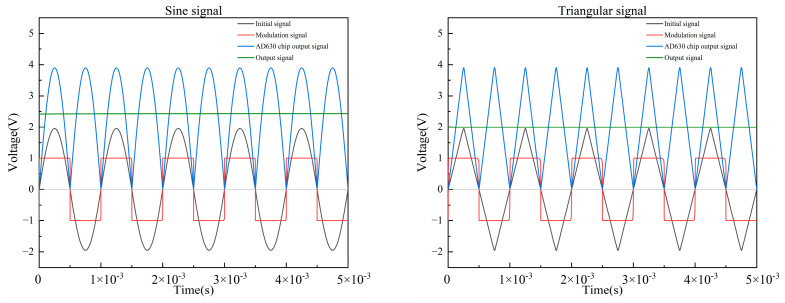

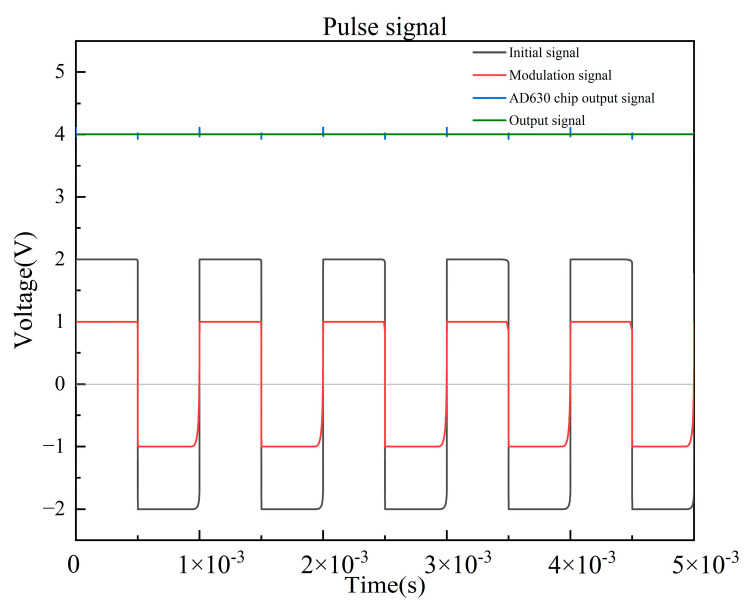
Demodulation principle of three excitation signals.

According to the working principle of the conditioning circuit, the sine signal, triangular signal, and pulse signal are analyzed, as shown in Figure 7. In Figure 7, the initial signal is the signal of the input conditioning module, and the output signal is the output signal of the conditioning module.

For the sine signals and the pulse signals, the modulation signal has the same frequency and the same phase as the initial signal, and the duty ratio of the pulse signal is 50%. When the modulation signal is positive, the positive voltage part of the initial signal is converted into a positive voltage with the same amplitude; when the modulation signal is negative, the negative voltage part of the initial signal is also converted into a positive voltage of the same amplitude. Finally, the initial signal is converted into the positive voltage half-wave signal, and the phase-locked processing of the signal is realized. For triangular signals, the modulation signal has the same frequency as the initial signal, but the phase lags the initial signal by 90°. In order to convert the triangular signal into a positive voltage half-wave signal, the lock phase processing of the signal can be realized only when the phase of the modulation signal lags behind the initial signal by 90°. Similarly, under the condition that the modulation signal and the initial signal have the same frequency, changing the phase relationship between them can realize the transformation of the initial signal into a negative voltage half-wave signal with the same amplitude.

### 5.2. Simulation Model of the Conditioning Circuit

**Figure 8:**
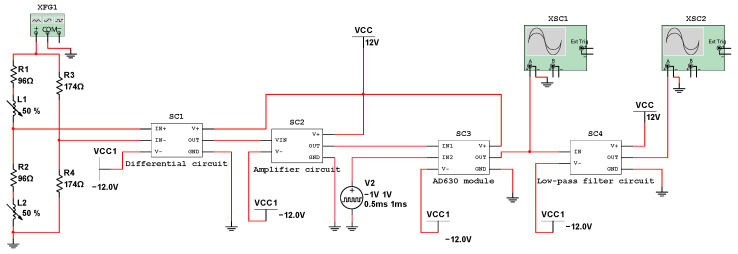
Simulation model of the conditioning circuit.

Multisim software was used to build the simulation model of the conditioning circuit, as shown in Figure 8. During the working process of the inductance coil, the resistance value is basically unchanged, and the equivalent resistance is 96 Ω. As the position of the sensing core changes, the inductance value changes, which is expressed by variable inductors. The differential circuit, the amplification circuit, the AD630 module, and the low-pass filter circuit are packaged into circuit modules. The magnification of the amplification circuit is 11 times. The signal generator XFG1 generates coil-excitation signals with an amplitude of 2 V and a frequency of 1000 Hz. The pulse voltage source V2 outputs the modulation signal to the AD630 module with an amplitude of 1 V and a period of 1 ms. The oscilloscopes XSC1 and XSC2 measure the output signal of the AD630 chip and the output signal of the low-pass filter, respectively.

The bridge circuit matching resistor *R_m_* is

(18) $$R_{m}=R+j\omega X_{L}=\sqrt{R^{2}+{\left(2\pi fL_{mid}\right)}^{2}}=174\Omega$$

where X_L_ is the inductive reactance of the coil, f is the frequency of the excitation voltage signal, and Lmid is the inductance of the coil when the sensing core is in the middle position.

### 5.3. Simulation Results and Analysis

**Figure 9:**
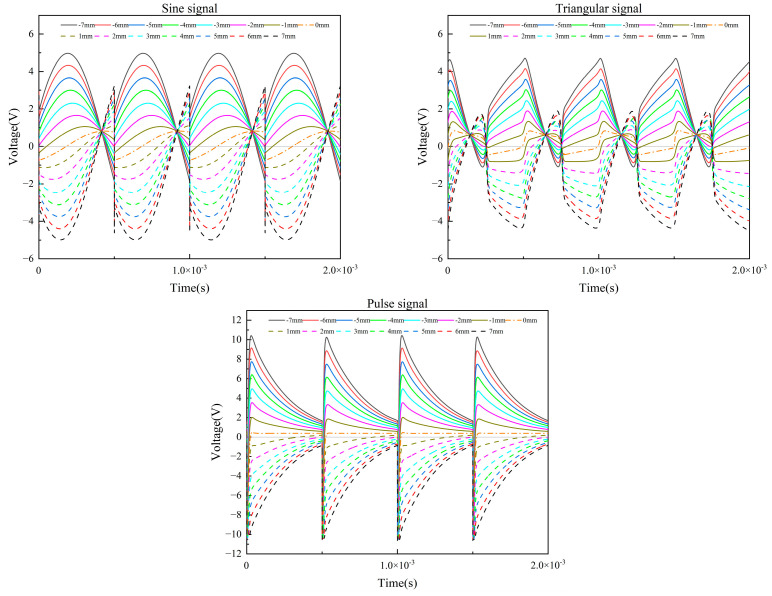
Output signal of the AD630 chip under three excitation signals.

Based on the simulation model of the conditioning circuit, it is necessary to measure the inductance of the coil when the sensing core is in different positions. DIS-14, a double coil inductance displacement transducer, is used in this study, and its measuring range is 14 mm. The inductance test device is made, which can measure the inductance of different displacements. After obtaining the inductance values for different displacements, the three excitation signals were simulated. When the sensing core is in different positions, the output signal of the AD630 chip is shown in Figure 9.

It can be seen from Figure 9 that the magnitude and direction of displacement can be tested by using the bridge circuit with three excitation signals. When the sensing core moves in the positive direction, the output voltage is negative; when the sensing core moves in the negative direction, the output voltage is positive. Under different excitation signals, the output signal of the AD630 chip is quite different in terms of amplitude change and phase shift.

During the entire stroke of the transducer, the maximum output voltage of the sine signal is 4.98 V, the minimum output voltage is −4.98 V, and the voltage range is 9.96 V. The maximum output voltage of the triangular signal is 4.70 V, the minimum output voltage is −4.37 V, and the voltage range is 9.07 V. The maximum output voltage of the pulse signal is 10.24 V, the minimum output voltage is −9.55 V, and the change range is 19.79 V. It can be seen that the voltage range of the pulse signal is the largest, and the voltage ranges of the sine signal and the triangular signal are similar. Therefore, when the displacement of the sensing core is the same, the voltage change of the pulse signal is the largest, and the sensitivity is the highest. In terms of sensitivity, a pulse signal is superior to a sine signal and a triangular signal.

The phase shift of the transducer has a great influence on the modulation signal and the output signal. When designing the conditioning circuit, the modulation signal should have good phase-locked processing capability for the initial signal. Normally, the frequency and phase of the modulation signal will not change during operation. When the initial signal has a phase shift, the modulation signal and the initial signal will be asynchronous, which will reduce the phase-locked processing ability of the conditioning circuit and the amplitude of the output signal. In Figure 9, the phase shift at the peak is taken as the phase shift of the signal. The phase shift was measured every 1 mm within the stroke of the sensing core, and the phase shift of the three excitation signals is shown in Table 1.

It can be seen from the table that, for sine signals, when the sensing core is displaced at the middle position, the maximum phase shift per unit displacement is 44.65°. The farther away from the middle position, the smaller the phase shift per unit displacement, and the average phase shift is 11.53°. The unit displacement phase shift of the triangle signal is small, and the average phase shift is 1.38°. The unit displacement phase shift of the pulse signal is the smallest, and the average phase shift is 0.73°. It can be seen that the phase shift of the sine signal is the largest, and the requirements for the modulation signal are the highest. For the signal with phase shift, in order to improve the phase-locked processing ability of the modulation signal relative to the initial signal, it is necessary to add a phase adjustment circuit. On the one hand, the phase of the initial signal can be adjusted. On the other hand, the phase of the modulation signal can be adjusted. Both of them can synchronize the phases of the modulation signal and the initial signal so that the conditioning circuit has good signal phase-locked processing capability. The phase shift of the triangular signal and the pulse signal is very small. Even without adding a phase adjustment circuit, better signal phase-locked processing can be achieved. Therefore, triangle and pulse signals are superior to sine signals in terms of phase shift.

**Figure 10:**
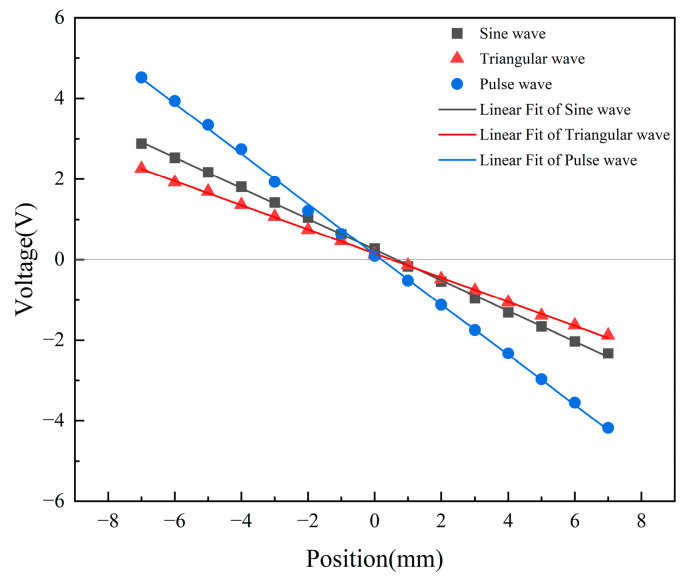
Output voltage at different positions.

The output signal of the low-pass filter is the final output signal of the displacement transducer, which is the DC voltage signal. The output voltage is measured once every 1 mm movement of the sensing core, and the collected data points are linearly fitted [38]. The results are shown in Figure 10. Table 2 shows the linear fitting parameters.

According to Figure 10 and Table 2, under the condition of linear fitting without weight, the intercept of the sine signal is the largest, the triangular signal is the second, and the pulse signal is the smallest. It shows that the sine signal has a large residual voltage at zero, which has a great impact on measurement accuracy and stability. The residual voltage at zero of the triangle signal and the pulse signal is smaller than the sine signal, so their measurement accuracy and stability are better than the sine signal. The slope of the pulse signal is the largest, followed by the sine signal, and the triangular signal is the smallest. This indicates that, within the stroke range, the output voltage of the pulse signal has the largest variation range and the highest sensitivity [39]. The sensitivity of the sine signals, the triangle signals, and the pulse signals is 0.3798 V/mm, 0.3084 V/mm, and 0.6473 V/mm, respectively. It can be seen from Pearson’s r and R-Square that the fitting effect of the triangular signal is the best, indicating that the displacement of the triangular signal has the greatest linear correlation with the output voltage. The linearities of the sine signal, the triangle signal, and the pulse signal are 1.50%, 1.47%, and 1.74%, respectively. Therefore, the triangle signal is superior to the sine and pulse signals in terms of linearity.

## 6. Experiment

**Figure 11:**
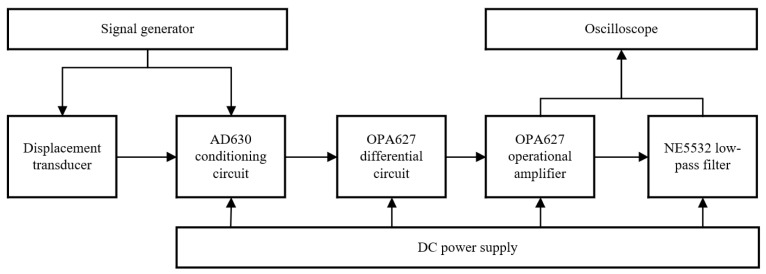
Experimental test block diagram.

**Figure 12:**
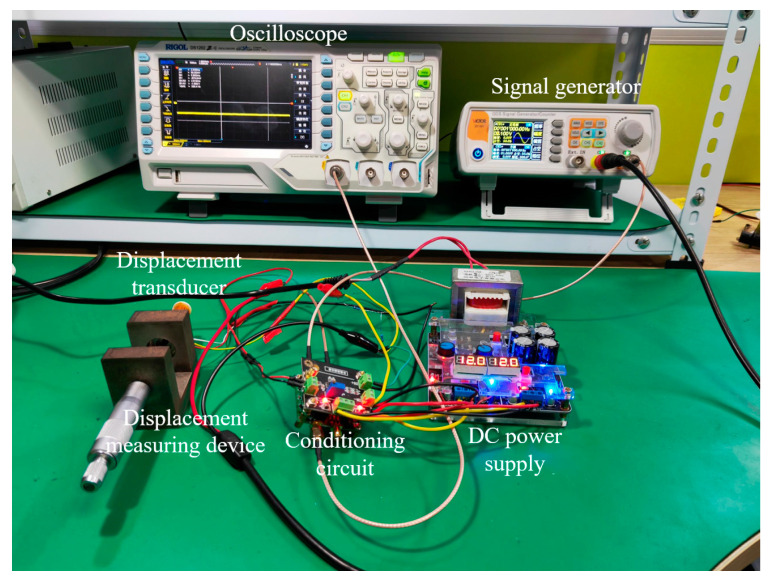
Test circuit.

Based on the above theoretical analysis and simulation model, a signal conditioning circuit board was made, and the experimental test block diagram and experimental circuit of the double-coil inductive displacement transducer were built, as shown in Figure 11 and Figure 12. The DC power supply is used for the power supply of the circuit board. The signal generator VICTOR2015H is used to generate the excitation voltage signal of the coil and the modulation signal of the conditioning circuit and can generate a sine voltage signal, a triangular voltage signal, and a pulse voltage signal. The maximum output frequency of VICTOR2015H is 15 MHz, the wavelength adoption rate is 266 MSa/s, and the output voltage amplitude is 2 mV–20 V. The frequency of the excitation signal from the experiment and simulation is the same, both at 1000 Hz. The displacement measuring device is composed of two parts: the transducer fixed bracket and the micrometer, wherein the micrometer can precisely control the movement and displacement of the sensing core. The conditioning circuit is the core of signal processing and includes four parts: the differential circuit, the amplifier circuit, the AD630 phase-locked circuit, and the low-pass filter circuit. The oscilloscope DS1202 has a real-time sampling rate of 1 GSa/s and a resolution of 12 bits, and it is used to measure the output signal of the conditioning circuit.

**Figure 13:**
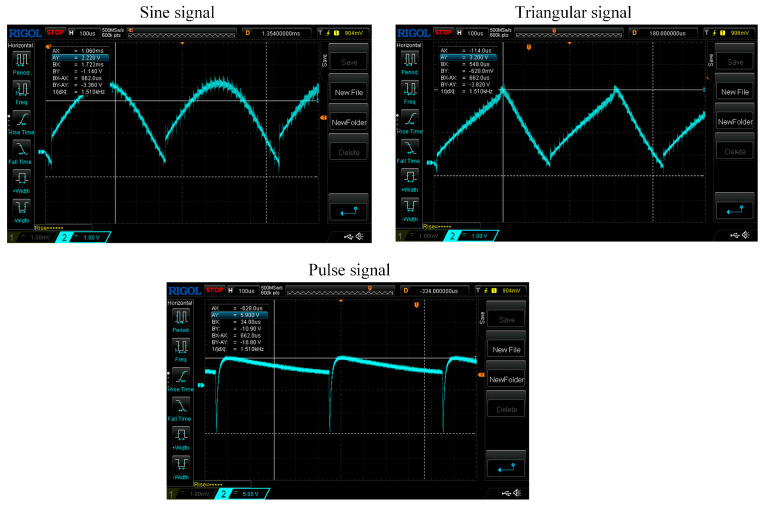
Experimental test of the output signal of the AD630 chip under 3 excitation signals.

Through the built test circuit, the actual signal waveforms of the AD630 output signal under 3 excitation signals were tested at the sensing core of −4 mm, as shown in Figure 13. As can be seen from the figure, the amplitudes of the three signals are basically the same as the simulation results. Sinusoidal and triangular signals have signal interference, while pulse signals have little interference. The results show that, compared with the other two excitation signals, pulse signals can suppress the interference of the conditioning circuit and work more reliably.

**Figure 14:**
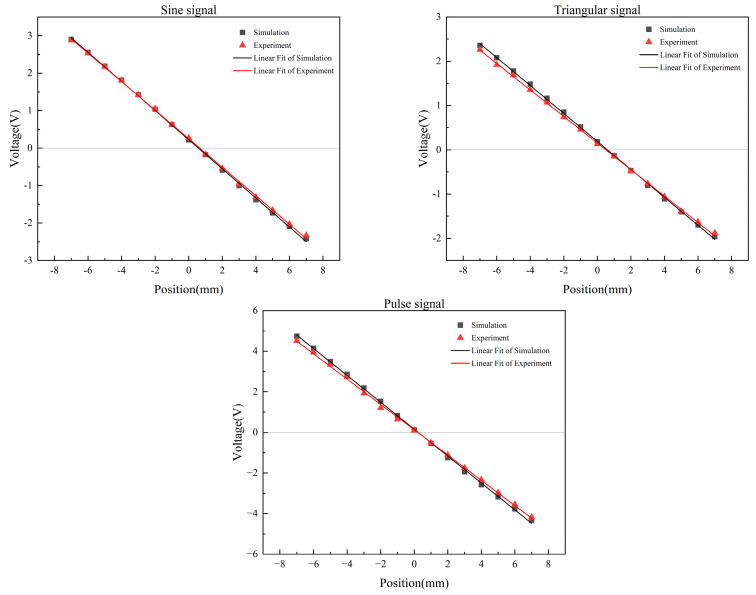
Comparison of simulation and test under three excitation signals.

Through the test circuit, the output voltages of the three excitation signals are tested and compared with the simulation results, as shown in Figure 14.

The comparison shows that under the excitation of three signals, the test value is slightly less than the simulation value, but the overall trend is consistent. According to the difference between experiment and simulation, the average error, linearity, and sensitivity of the output voltage are analyzed.

In terms of average error, the sine signal is 4.02%, the triangle signal is 7.21%, the pulse signal is 9.85%, and the overall average error is 7.03%. The average error of the experiment and simulation is within 10%, which is acceptable. In terms of linearity, the sine signal is 1.61%, the triangle signal is 1.56%, and the pulse signal is 1.95%. Compared with the simulation results, the error of the sine signal is 3.21%, the error of the triangle signal is 6.12%, and the error of the pulse signal is 12.07%. The average linearity error of the three signals is 7.13%, which is within the reasonable error range. In terms of sensitivity, the sine signal is 0.372 V/mm, the triangle signal is 0.300 V/mm, and the pulse signal is 0.621 V/mm. Compared with the simulation results, the error of the sine signal is 2.05%, the error of the triangle signal is 2.72%, and the error of the pulse signal is 4.06%. The average sensitivity error of the three signals is 2.94%, which is within the reasonable error range.

The difference between experiment and simulation includes the following aspects: First, the displacement transducer of the simulation circuit is an equivalent model, and there is an error with the actual circuit. Second, the signal source in the simulation circuit is an ideal standard signal, but the actual circuit is not. Third, the real value of electronic components in the simulation circuit is the same as the nominal value, but there is an error between the real value and the nominal value of electronic components in the actual circuit. Fourth, the actual circuit is connected by wires, which will produce resistance and parasitic capacitance. The layout of the circuit board will also produce parasitic inductance and parasitic capacitance. Both will have an effect on the output signal. Fifth, there is an external interference signal in the actual circuit, and the test environment will affect the output signal. Sixth, during the experiment, researchers will have measurement errors, which will lead to the difference between the experiment and the simulation.

In general, although there are certain differences between the experiment and the simulation, the error is within a reasonable range. The effectiveness of the two coil excitation signals was verified by experiments.

## 7. Conclusions

In this paper, the influence of the coil excitation signal on the performance of a double-coil inductive displacement transducer is studied. The working principle and characteristics of the double-coil inductive displacement transducer are analyzed, and the signal conditioning circuit is designed based on the AD630 chip. The signal from the transducer is demodulated by using the bridge circuit and conditioning circuit. Two coil excitation signals are proposed, which are generated directly by the microprocessor. The demodulation principles of three excitation signals are analyzed. The simulation model of two coil inductive displacement transducers is established, and the effects of three excitation signals on the performance are compared and analyzed. The effectiveness of the scheme has been verified by experiments. The following conclusions have been drawn from the study of this paper:

(1) For the double-coil inductive displacement transducer, when the length-to-diameter ratio of the coil is *l/r* ≫ 1, there is a linear relationship between the output signal and the displacement of the sensing core. In practical applications, the length-to-diameter ratio of the coil is not infinite, so the output signal and the displacement of the sensing core are approximately linear;
(2) Based on the bridge circuit and signal conditioning circuit, the output signal of the double-coil inductive displacement transducer can be demodulated by adjusting the frequency and phase of the excitation signal and the modulation signal;
(3) The three excitation signals have different effects on the performance of the double-coil inductive displacement transducer. In terms of phase shift, the triangular and pulse signals are superior to the sine signals. In terms of sensitivity, the pulse signal is superior to the sine and triangular signals. In terms of linearity, the triangle signal is superior to the sine and pulse signals;
(4) Compared with the commonly used sine excitation signals, the proposed two excitation signals can improve the performance of the double-coil inductive displacement transducer. In different applications, the triangular signal and the pulse signal can replace the triangular signal. The triangular and pulse excitation signals can replace sine excitation signals for different performance requirements.

The microprocessor in the controller can directly generate the triangle signal and the pulse signal. Compared with the sine signal, using the triangular and pulse signals reduces the sine signal processing circuit and production costs. In terms of transducer performance, both the triangle signal and the pulse signal are better than the traditional sine signal. Generally speaking, the two coil excitation signals proposed in this paper are superior to traditional excitation signals and can replace the traditional excitation signals.

## Figures and Tables

**Table 1:** Phase shift of three excitation signals.

Displacement Change	Phase Shift (deg)		
	Sine Signal	Triangular Signal	Pulse Signal
+6 mm → +7 mm	0.66	0.16	−0.33
+5 mm → +6 mm	2.63	1.64	−0.98
+4 mm → +5 mm	2.46	−4.76	−0.33
+3 mm → +4 mm	4.76	7.22	0.00
+2 mm → +3 mm	9.03	0.66	−0.98
+1 mm → +2 mm	19.21	−1.15	−1.15
0 → +1 mm	36.28	1.64	−1.31
0 → −1 mm	−44.65	−3.78	−0.33
−1 mm → −2 mm	−20.68	−2.46	−0.98
−2 mm → −3 mm	−10.34	−1.64	−0.33
−3 mm → −4 mm	−4.92	−1.64	0.00
−4 mm → −5 mm	−1.97	−0.82	−0.98
−5 mm → −6 mm	−2.63	−0.66	−1.15
−6 mm → −7 mm	−1.15	−0.66	−1.31

**Table 2:** Linear fitting parameters of output voltage.

Equation	y = a + b*x		
Plot	Sine signal	Triangular signal	Pulse signal
Weight	No Weighting		
Intercept	0.22493 ± 0.01051	0.18893 ± 0.0083	0.15847 ± 0.01877
Slope	−0.38853 ± 0.00243	−0.31564 ± 0.00192	−0.66242 ± 0.00434
Pearson’s r	−0.99975	−0.99976	−0.99972
R-Square (COD)	0.99949	0.99952	0.99944

## Data Availability

The data presented in this study are available on request from the corresponding author.
